# Comment on Takakura et al. Acupuncture for Japanese Katakori (Chronic Neck Pain): A Randomized Placebo-Controlled Double-Blind Study. *Medicina* 2023, *59*, 2141

**DOI:** 10.3390/medicina60071030

**Published:** 2024-06-24

**Authors:** Stephen Birch, Terje Alraek, Tae-Hun Kim, Myeong Soo Lee

**Affiliations:** 1 School of Health Sciences, Kristiania University College, 0107 Oslo, Norway; terje.alrak@uit.no; 2 The National Research Center in Complementary and Alternative Medicine (NAFKAM), Department of Community Medicine, Faculty of Health Science, UiT, The Arctic University of Norway, 9037 Tromsø, Norway; 3 Korean Medicine Clinical Trial Center, Korean Medicine Hospital, Kyung Hee University, Seoul 02447, Republic of Korea; rockandmineral@gmail.com; 4 KM Science Research Division, Korea Institute of Oriental Medicine, Daejeon 34054, Republic of Korea; drmslee@gmail.com

## Methods Matter

We are writing because we have a number of concerns about the Takakura et al. acupuncture trial on shoulder pain [1].

When acupuncture is tested in clinical trials that attempt to control for placebo using sham acupuncture, two key variables are controlled for: the sites of needling and the techniques of needling [2]. If the research question concerns the effectiveness of a needling technique, both the test acupuncture and sham acupuncture techniques are applied to the same loci; if the research question concerns the effectiveness of the loci of needling, both the test loci and sham loci receive the same needling technique [3,4,5]. In both models, provided that randomization and blinding are successfully applied, placebo is kept equal between the treatment groups [2] and neither are placebo-controlled trials testing the efficacy of acupuncture, since two active treatments are compared to each other, rather than comparing a test treatment to a placebo treatment [2,5,6]. To test the effectiveness of acupuncture against a putative placebo, both the technique of needling and the sites of needling in the sham must be different [2], and the sham technique must demonstrate a lack of specific effects [5].

In the trial by Takakura and colleagues [1], two sham techniques were used in two treatment arms: one using a non-penetrating sham where the blunt needle tip touched the skin and one where the non-penetrating sham needle did not touch the skin. Both used special mounts to hold the needles and insertion tubes in place, and both applied the treatment techniques to the same acupoints as in the test acupuncture. While studies have demonstrated that sham techniques could serve as placebo-controlled treatments by demonstrating successful blinding [7,8], this trial cannot be considered a placebo-controlled trial since it applied the research methods used to compare the needling techniques with placebo kept equal between the groups—the different needling techniques were all applied to the same acupoints. It was a comparison of treatment techniques and not a placebo-controlled trial testing the efficacy of acupuncture. Since the trial found a significant improvement in all three acupuncture treatment arms compared to the no-acupuncture arm, the trial demonstrated that, for the treatment of the shoulder pain targeted by the trial, it is not necessary to insert needles for the treatment to be effective. There are a number of non-insertion needling techniques and systems in acupuncture [2,9,10], and this trial helps to demonstrate the potential use of these in the treatment of shoulder pain.

Other trialists have made the same mistakes as this trial, applying the sham techniques to the same acupoints, further, a systematic review and meta-analysis of similarly designed trials demonstrated that non-penetrating sham techniques are effective, with specific effects on the conditions treated [6]. There are plausible and established biological mechanisms by which non-invasive sham techniques might work. A recent systematic review found that sham acupuncture techniques often trigger the same physiological effects as the test acupuncture, influencing multiple biomarkers [11], with a range of other potential mechanisms triggered by the sham techniques [12]. 

Unfortunately, the trialists conducted this trial with the intention of controlling for placebo effects and attempting to separate different aspects of the placebo effects, including the ritual of the therapy: “Four hundred patients…. were randomly assigned to penetrating needle treatment (acupuncture ritual and skin penetration), skin-touch needle treatment (acupuncture ritual and skin touch), no-touch needle treatment (acupuncture ritual alone)....” [1]. The methodology used in the trial was incapable of accomplishing the goals of the study, namely to compare acupuncture to a placebo and identify the components of the placebo effect, such as the ritual of the therapy. The conclusions drawn by the researchers about the potential role of the placebo and the ritual of the therapy are invalid and do not follow from this trial. This trial accidentally included biologically active components of the acupuncture treatment in the sham treatments, making them potentially effective in their own rights and risking the underestimation of the acupuncture treatment’s effects [5]. The accidental inclusion of active components in a placebo treatment capable of triggering specific effects has been identified as a significant problem in placebo-controlled trials and is more common than had been previously thought [13,14,15]. The inadequate description of sham acupuncture techniques [16] and the lack of pre-clinical studies demonstrating the lack of specific effects with sham needling techniques [5] continue to plague this field, creating difficulties in interpreting the results from both clinical trials and systematic reviews when sham acupuncture is used as a control treatment [5].
